# MYC overexpression but not MYC/BCL2 double expression predicts survival in bulky mass diffuse large B‐cell lymphoma patients

**DOI:** 10.1002/cam4.6463

**Published:** 2023-08-28

**Authors:** Yanjie Wang, Donglin Liu, Xudong Zhang, Mingzhi Zhang, Shenglei Li, Xiaoyan Feng, Meng Dong, Shanshan Ma, Siyu Qian, Zeyuan Wang, Yue Zhang, Pengyuan Wang, Shuhao Mei, Qingjiang Chen

**Affiliations:** ^1^ Department of Oncology, Henan Province Lymphoma Treatment Center The First Affiliated Hospital of Zhengzhou University Zhengzhou China; ^2^ Department of Pathology The First Affiliated Hospital of Zhengzhou University Zhengzhou China; ^3^ Department of Medical Oncology Xuchang Central Hospital Xuchang China; ^4^ Department of Hematology Xuchang Central Hospital Xuchang China

**Keywords:** diffuse large B‐cell lymphoma, bulky mass, immunohistochemistry, lymphoma, MYC, radiotherapy

## Abstract

**Purpose:**

The prognostic factors for diffuse large B‐cell lymphoma (DLBCL) have been fully explored, but prognostic information for bulky mass DLBCL patients is limited. This study aimed to analyze the prognostic value of MYC protein expression and other biological parameters in bulky mass DLBCL patients.

**Methods:**

We defined a bulky mass as a maximum tumor diameter ≥7.5 cm and studied 227 patients with de novo bulky mass DLBCL.

**Results:**

In all patients with bulky mass DLBCL, the 1‐year and 3‐year OS rates were 72.7% and 57.1%, respectively, and the 1‐year and 3‐year PFS rates were 52.0% and 42.5%, respectively. The MYC overexpression group (*n* = 140) showed significantly worse overall survival (OS; *p* = 0.019) and progression‐free survival (PFS; *p* = 0.001) than the non‐MYC overexpression group (*n* = 87). Subgroup analyses demonstrated that the MYC overexpression group was associated with inferior OS and PFS in the subgroups with the International Prognostic Index score of 3–5 (OS: *p* = 0.011; PFS: *p* < 0.001), Ann Arbor stage 3–4 (OS: *p* = 0.014; PFS: *p* < 0.001) and GCB subtype (OS: *p* = 0.014; PFS: *p* = 0.010). Consolidation radiotherapy improved OS and PFS in patients with bulky mass DLBCL (OS: *p* = 0.008; PFS: *p* = 0.004) as well as in those with MYC overexpression (OS: *p* = 0.001; PFS: *p* = 0.001). The prognostic value of MYC overexpression was maintained in a multivariate model adjusted for the International Prognostic Index.

**Conclusion:**

MYC overexpression is a poor predictor for bulky mass DLBCL patients. Consolidation radiotherapy for residual disease after induction therapy may improve outcomes for patients with bulky mass DLBCL.

## Graphical abstract

Our study showed that MYC overexpression but not MYC/BCL2 double expression is a poor predictor for bulky mass DLBCL patients. Consolidation radiotherapy for residual disease after induction therapy may improve outcomes for patients with bulky mass DLBCL.

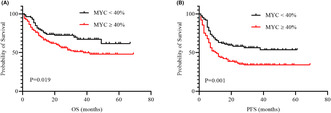

## 1 INTRODUCTION

Diffuse large B‐cell lymphoma (DLBCL) is a heterogeneous disease with distinct clinical, histological, and molecular characteristics. Rituximab combined with cyclophosphamide, hydroxydaunorubicin, vincristine, and prednisone (R‐CHOP) is recognized as the standard therapy. However, up to 40% of DLBCL patients exhibit refractory disease or disease relapse. Therefore, it is crucial to identify DLBCL subgroups with a poor prognosis. The International Prognostic Index (IPI) is commonly used to stratify the risk prognosis of DLBCL, but it does not capture biological parameters, which may lead to inaccurate risk stratification. An increasing number of studies have demonstrated the prognostic effect of MYC and BCL2 gene or protein levels. The MYC gene is located on chromosome 8q24 and has been reported to regulate up to 10% of genes within the human genome.^1^ The deregulation of MYC drives many carcinogenic processes involving proliferation, differentiation, and metabolism.^2^, ^3^, ^4^ Notably, DLBCL patients with MYC/BCL2 double expression (DE) have been reported to have significantly poor survival.^5^, ^6^, ^7^, ^8^, ^9^ Therefore, the prognostic role of biological parameters in DLBCL should be underlined.

Moreover, DLBCL patients with bulky mass are recognized as a distinct group with inferior survival outcomes.^10^, ^11^, ^12^ However, the prognostic role of clinical and biological parameters in patients with bulky mass DLBCL has been little explored. Therefore, this study aimed to investigate the effect of biological parameters for bulky mass DLBCL patients and further explore the prognostic value of different treatment strategies for bulky mass DLBCL.

## 2 MATERIALS AND METHODS

### 2.1 Patients

We reviewed formalin‐fixed, paraffin‐embedded (FFPF) biopsies from 227 patients with de novo bulky mass DLBCL at the First Affiliated Hospital of Zhengzhou University between February 2018 and December 2021. The diagnosis of DLBCL was based on the 2008 WHO classification criteria.^13^ A bulky mass was defined as a maximum tumor diameter (MTD) ≥7.5 cm. Patients with the following were excluded: (1) primary central nervous system lymphoma; (2) primary mediastinal large B‐cell lymphoma; and (3) DLBCL transformed from low‐grade B‐cell lymphoma. Clinical data were obtained by consulting hospitalization records and through telephone interviews. All patients received R‐CHOP or intensive‐dose therapy such as rituximab, etoposide, prednisone, cyclophosphamide, and doxorubicin (R‐EPOCH) as the first‐line induction therapy. Those without appropriate clinical information were excluded.

### 2.2 Immunohistochemistry (IHC) and FISH

**FIGURE 1:**
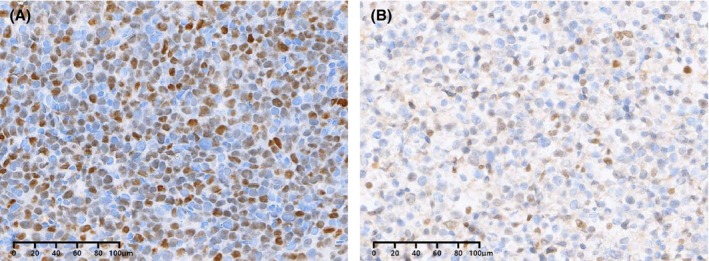
MYC protein expression measured by immunohistochemistry in bulky mass DLBCL. MYC staining pattern is nuclear. (A) MYC protein expression ≥40%; (B) MYC protein expression <40%.

All tissues were fixed in 3.7% neutral formaldehyde, routinely dehydrated, paraffin‐embedded, and serially sectioned to 2–3 μm thick for H&E and immunohistochemical staining. The EnVision two‐step method was used for immunohistochemistry. Monoclonal antibodies against MYC (MXB, Fuzhou, China), BCL2 (ZSGB, Beijing, China), BCL6 (ZSGB), and Ki‐67 (Gene Tech, Shanghai, China) were used. The cutoff scores defined as protein overexpression were 40% for MYC (Figure 1), 50% for BCL2, and 50% for BCL6, as reported in previous studies.^7^, ^14^, ^15^, ^16^, ^17^, ^18^ The cell‐of‐origin (COO) subtypes based on the HANS algorithm were classified as GCB and non‐GCB and were determined by the immunohistochemical expression of CD10, BCL6, and MUM1.

FISH analysis for MYC, BCL2, and BCL6 was performed based on a tissue microarray (TMA) using dual‐colour break‐apart probes (Abbott Molecular, Des Plaines, IL, USA). The signals from 100 interphase nuclei were analyzed. The cases with break‐apart signals >10% of nuclei were considered positive for the presence of a translocation.

### 2.3 Statistical analysis

Overall survival (OS) was measured from the date of diagnosis until death of any cause or the last follow‐up. Progression‐free disease (PFS) was measured from the date of diagnosis until the date of disease progression, relapse, or death of any cause. Response assessment was defined as complete response (CR), partial response (PR), stable disease (SD), and progressive disease (PD).^19^ The overall response rate (ORR) was defined as the proportion of patients who achieved CR or PR to therapy. Survival analyses were performed with the Kaplan–Meier method and were compared using the log‐rank test. Pearson's χ^2^ test or Fisher's exact test was used to assess differences in categorical variables. Univariate and multivariate analyses were performed using the Cox proportional hazards regression model. Statistical analysis was performed using SPSS version 23.0. A *p* value of <0.05 was considered statistically significant.

## 3 RESULTS

### 3.1 Clinicopathological characteristics

The baseline characteristics of patients with bulky mass DLBCL are presented in Table 1. There were 91 females and 136 males with a median age of 56 years (range, 18–89). A total of 89 (39.2%) patients were 60 years or older. A total of 159 (70.0%) patients had ≥2 extranodal sites of disease. Twenty‐five (11.5%) patients had bone marrow involvement, and 13 (5.8%) had central nervous system involvement by lymphoma. A total of 164 (72.2%) patients had stage 3–4 disease according to the Ann Arbor staging classification, and 125 (55.3%) had an IPI score of 3–5. Sixty‐six (29.1%) patients had comorbidities, such as hypertension, coronary heart disease, and cerebrovascular disease.

**TABLE 1:** Clinicopathologic features of bulky mass DLBCL according to MYC expression.

Characteristics	Total (*n* = 227)	Non‐MYC overexpression (*n* = 87)	MCY overexpression (*n* = 140)	*p* Value
Sex				
Female	91 (40.1%)	38 (43.7%)	53 (37.9%)	0.384
Male	136 (59.9%)	49 (56.3%)	87 (62.1%)	
Age				
<60	138 (60.8%)	58 (66.7%)	80 (57.1%)	0.153
≥60	89 (39.2%)	29 (33.3%)	60 (42.9%)	
Performance status				
ECOG <2	132 (58.1%)	55 (63.2%)	77 (55.0%)	0.222
ECOG ≥2	95 (41.9%)	32 (36.8%)	63 (45.0%)	
Ann Arbor stage				
1–2	63 (27.8%)	30 (34.5%)	33 (23.6%)	0.074
3–4	164 (72.2%)	57 (65.5%)	107 (76.4%)	
IPI				
0–2	101 (44.7%)	39 (44.8%)	62 (44.6%)	0.974
3–5	125 (55.3%)	48 (55.2%)	77 (55.4%)	
NA	1	0	1	
COO				
GCB	63 (28.1%)	23 (27.4%)	40 (28.6%)	0.848
Non‐GCB	161 (71.9%)	61 (72.6%)	100 (71.4%)	
NA	3	3	0	
Extranodal sites ≥2				
NO	68 (30.0%)	30 (34.5%)	38 (27.1%)	0.240
YES	159 (70.0%)	57 (65.5%)	102 (72.9%)	
B symptom				
NO	152 (67.0%)	62 (71.3%)	90 (64.3%)	0.277
YES	75 (33.0%)	25 (28.7%)	50 (35.7%)	
BM involvement				
NO	192 (88.5%)	76 (91.6%)	116 (86.6%)	0.262
YES	25 (11.5%)	7 (8.4%)	18 (13.4%)	
NA	10	3	7	
CNS involvement				
NO	210 (94.2%)	81 (94.2%)	129 (94.2%)	0.994
YES	13 (5.8%)	5 (5.8%)	8 (5.8%)	
NA	4	1	3	
MTD				
<10 cm	113 (49.8%)	47 (54.0%)	66 (47.1%)	0.313
≥10 cm	114 (50.2%)	40 (46.0%)	74 (52.9%)	
LDH				
Normal	31 (14.0%)	14 (16.7%)	17 (12.3%)	0.365
Elevated	191 (86.0%)	70 (83.3%)	121 (87.7%)	
NA	5	3	2	
β2‐MG				
Normal	132 (59.8%)	54 (63.5%)	78 (56.5%)	0.301
Elevated	91 (40.8%)	31 (36.5%)	60 (43.5%)	
NA	0	2	2	
MYC translocation				
NO	164 (93.7)	59 (96.7%)	105 (92.1%)	0.231
YES	11 (6.3%)	2 (3.3%)	9 (7.9%)	
NA	52	26	26	
BCL2 translocation				
NO	165 (94.8%)	59 (96.7%)	106 (93.8%)	0.407
YES	9 (5.2%)	2 (3.3%)	7 (6.2%)	
NA	53	26	27	
BCL6 translocation				
NO	130 (74.7%)	47 (77.0%)	83 (73.5%)	0.602
YES	44 (25.3%)	14 (23.0%)	30 (26.5%)	
NA	53	26	27	

Abbreviations: BM, bone marrow; CNS, central nervous system; COO, cell‐of‐origin; GCB, Germinal Center B‐cell like; IPI, International Prognostic Index; LDH, lactic dehydrogenase; MTD, maximum tumor diameter; NA, not available; OS, overall survival; PFS, progression‐free survival; β2‐MG, β2‐microglobulin.

All patients (*n* = 227) received immunochemotherapy as the first‐line induction treatment: 180 (79.3%) patients received R‐CHOP, and 47 (20.7%) patients received R‐EPOCH. Of these 227 patients, 14 (6.2%) patients underwent surgery before induction treatment, 37 (16.3%) patients underwent consolidation radiotherapy (RT) for the residual disease after induction treatment, and 10 (4.4%) received autologous hematopoietic stem cell transplantation after CR was achieved. The baseline characteristics were similar between the non‐MYC overexpression (*n* = 87) group and MYC overexpression (*n* = 140) group.

### 3.2 MYC overexpression status and survival outcome

**FIGURE 2:**
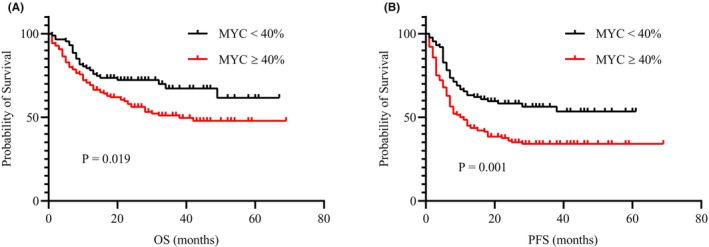
Survival analysis according to MYC expression in the entire cohort. (A) OS and (B) PFS.

In all patients with bulky mass DLBCL, the 1‐year and 3‐year OS rates were 72.7% and 57.1%, respectively, and the 1‐year and 3‐year PFS rates were 52.0% and 42.5%, respectively, with a median follow‐up duration of 23 months (range, 1–69). All patients (*n* = 227) had MYC status confirmed by immunohistochemistry at the time of initial diagnosis. A total of 140 of 227 (61.7%) patients had MYC overexpression. The MYC overexpression group had a significantly worse OS than the non‐MYC overexpression group, with 3‐year OS rates of 51.1% and 67.3%, respectively (*p* = 0.019; Figure 2A). Similarly, patients with MYC overexpression demonstrated a significantly worse PFS than those with non‐MYC overexpression, with 3‐year PFS rates of 34.1% and 56.3%, respectively (*p* = 0.001; Figure 2B).

Moreover, compared with the non‐MYC overexpression group, patients with MYC overexpression exhibited a worse ORR to first‐line induction treatment (*p* = 0.005, 69.0% vs. 50.0%). As of the date of follow‐up, a greater proportion of patients in the MYC overexpression group compared with the non‐MYC overexpression group experienced disease relapse and progression (*p* = 0.002, 31.0% vs. 52.1%).

### 3.3 Survival outcomes according to MYC overexpression status in the IPI, stage, and COO subtype subgroups

**FIGURE 3:**
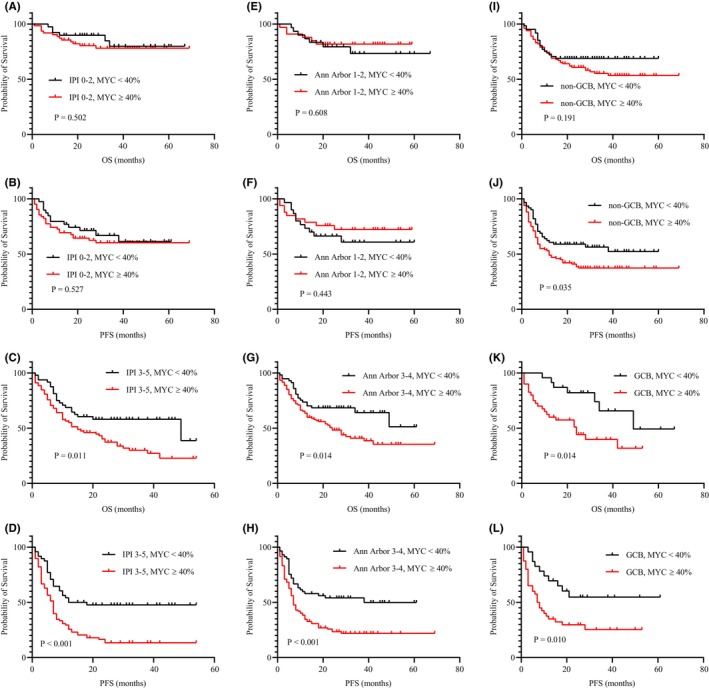
Survival analysis according to MYC expression in different subgroups. (A, B) OS and PFS in IPI score of 0–2 subgroup; (C, D) OS and PFS in IPI score of 3–5 subgroup; (E, F) OS and PFS in Ann Arbor stage 1–2 subgroup; (G, H) OS and PFS in Ann Arbor stage 3–4 subgroup; (I, J) OS and PFS in non‐GCB subgroup; (K, L) OS and PFS in GCB subgroup.

Further survival analysis was investigated according to the MYC overexpression status based on the IPI, stage, and COO subtype subgroups. MYC overexpression status was significantly associated with inferior OS and PFS in the subgroup with IPI score of 3–5 (OS; *p* = 0.011, PFS; *p* < 0.001; Figure 3C, D) and in the Ann Arbor stage 3–4 subgroup (OS; *p* = 0.014, PFS; *p* < 0.001; Figure 3G, H) but not in the subgroup with IPI score of 0–2 or in the Ann Arbor stage 1–2 subgroup (*p* > 0.05; Figure 3A, B, E, F). In addition, MYC overexpression status was significantly associated with inferior OS in patients with the GCB subtype (*p* = 0.014; Figure 3K) but not in those with the non‐GCB subtype (*p* = 0.191; Figure 3I). Inferior PFS was observed in both patients with the GCB subtype (*p* = 0.010; Figure 3L) and those with the non‐GCB subtype (*p* = 0.035; Figure 3J). Furthermore, compared with the non‐MYC overexpression group, the MYC overexpression group exhibited a worse ORR and higher disease relapse and progression rates in the subgroups with IPI score of 3–5, Ann Arbor stage 3–4, and GCB subtype (*p* < 0.05) but not in the subgroups with IPI score of 0–2, Ann Arbor stage 1–2, and non‐GCB subtype (*p* > 0.05).

### 3.4 Prognostic significance between MYC expression and different treatment strategies

With respect to the impact of various treatment strategies on survival among bulky mass DLBCL patients, we did not observe a significant difference in survival outcomes between patients who did or did not receive dose‐intensive immunohistochemistry treatment such as R‐EPOCH, surgery, and autologous transplantation. By contrast, consolidation RT was associated with a better OS (*p* = 0.008) and PFS (*p* = 0.004) in all patients with bulky mass DLBCL. Of pivotal importance, we identified that RT was associated with a preferable OS (*p* = 0.001) and PFS (*p* = 0.001) in patients with MYC overexpression. However, RT showed no significant differences in OS (*p* = 0.704) and PFS (*p* = 0.523) within the non‐MYC overexpression group.

### 3.5 DE and DH status and survival outcomes

BCL2 overexpression was detected in 151 of 194 (77.8%) patients, BCL6 in 112 of 146 (76.7%) patients, and MYC/BCL2 DE in 106 of 194 (54.6%) patients. By contrast to the results of previous studies, compared with non‐MYC/BCL2 DE status, MYC/BCL2 DE status did not correlate with poorer OS (*p* = 0.318) and PFS (*p* = 0.114). The MYC, BCL2, and BCL6 cytogenetic statuses were confirmed by FISH in 175, 174, and 174 patients, respectively. MYC rearrangement was detected in 11 of 175 (6.3%) patients, BCL2 rearrangement in nine of 174 (5.2%) and BCL6 rearrangement in 44 of 174 (19.4%). MYC/BCL2 or MYC/BCL6 double hit (DH) was present in two of 173 (1.1%) patients. MYC rearrangement, BCL2 rearrangement, BCL6 rearrangement, and DH status did not correlate with a significantly worse OS and PFS (*p* > 0.05).

### 3.6 Univariate and multivariate analyses of survival outcomes

Potential prognostic factors for bulky mass DLBCL were analyzed in univariate and multivariate analyses. Univariate analysis demonstrated that age ≥60 years, Ann Abor stage 3–4, IPI score of 3–5, elevated β2‐microglobulin level, comorbidities and MYC overexpression showed significant associations with both a poor OS and a poor PFS (*p* < 0.05, Table 2). In the multivariate analysis, MYC overexpression had a significant prognostic impact on OS (vs. non‐MYC overexpression group; HR, 1.679; 95% CI, 1.049–2.688, *p* = 0.031) and PFS (vs. non‐MYC overexpression group; HR, 1.864; 95% CI, 1.255–2.770, *p* = 0.002) after adjusting for the IPI score (Table 3).

**TABLE 2:** Univariate analysis of OS and PFS of bulky mass DLBCL.

Variables	OS		PFS	
	HR (95% CI)	*p* Value	HR (95% CI)	*p* Value
Male (vs. female)	1.047 (0.693 ~ 1.580)	0.828	1.123 (0.788 ~ 1.600)	0.522
Age ≥60 years	2.822 (1.871 ~ 4.257)	<0.001	2.106 (1.489 ~ 2.978)	<0.001
Ann Arbor Stage 3–4	3.046 (1.692 ~ 5.483)	<0.001	2.893 (1.793 ~ 4.669)	<0.001
IPI 3–5	4.377 (2.636 ~ 7.270)	<0.001	2.850 (1.938 ~ 4.191)	<0.001
GCB (vs. non‐GCB)	1.275 (0.825 ~ 1.970)	0.274	1.203 (0.825 ~ 1.753)	0.337
LDH elevated	1.399 (0.725 ~ 2.700)	0.317	1.652 (0.930 ~ 2.935)	0.087
β2‐MG elevated	2.299 (1.513 ~ 3.492)	<0.001	2.169 (1.524 ~ 3.087)	<0.001
MTD ≥10 cm	1.246 (0.829 ~ 1.871)	0.290	1.372 (0.969 ~ 1.942)	0.074
Comorbidity	1.917 (1.266 ~ 2.904)	0.002	1.479 (1.027 ~ 2.129)	0.035
MYC/BCL2 DE	1.292 (0.848 ~ 1.969)	0.232	1.349 (0.922 ~ 1.972)	0.124
MYC overexpression	1.687 (1.079 ~ 2.637)	0.022	1.824 (1.249 ~ 2.664)	0.002

Abbreviations: CI, confidence interval; DE, double expression; HR, hazard ratio.

**TABLE 3:** Multivariate analysis of OS and PFS of bulky mass DLBCL.

Variables	OS		PFS	
	HR (95% CI)	*p* Value	HR (95% CI)	*p* Value
Age ≥60 years	1.324 (0.798 ~ 2.199)	0.277	1.146 (0.737 ~ 1.783)	0.546
Ann Arbor Stage 3–4	1.645 (0.849 ~ 3.226)	0.139	1.865 (1.093 ~ 3.183)	0.022
IPI score of 3–5	2.868 (1.527 ~ 5.386)	0.001	1.977 (1.202 ~ 3.250)	0.007
β2‐MG elevated	1.403 (0.902 ~ 2.182)	0.133	1.378 (0.941 ~ 2.019)	0.100
Comorbidity	1.444 (0.923 ~ 2.258)	0.108	1.168 (0.789 ~ 1.728)	0.438
MYC overexpression	1.679 (1.049 ~ 2.688)	0.031	1.864 (1.255 ~ 2.770)	0.002

## 4 DISCUSSION

Bulky mass is a significant adverse prognostic factor for DLBCL; however, studies regarding the effect of biological parameters on bulky mass DLBCL are limited. Therefore, this study aimed to supplement the data in this field.

Many studies have shown that MYC plays a pivotal role in the occurrence and development of lymphoma. MYC deregulation can occur at the level of gene transcription, mRNA stability, and protein modification.^20^ However, FISH can only be used to detect MYC rearrangement at the gene level. Some findings have suggested that MYC protein expression can also occur via alternative nontranslocation‐based mechanisms in lymphoma lacking rearrangement of the MYC gene.^7^, ^21^ For example, miRNAs may regulate MYC protein expression. Leucci et al. found that the miRNA hsa‐miR‐34b, which downregulated in lymphoma without an MYC rearrangement, was inversely related to MYC protein expression in a dose‐dependent manner.^22^ Onnis et al. identified another miRNA, hsa‐miR‐9, that positively controlled MYC protein expression in lymphoma without an MYC rearrangement.^23^ An increased MYC gene copy number has been shown to be associated with an increased MYC protein expression level in DLBCL.^24^, ^25^ Furthermore, an in vitro experiment demonstrated that MYC protein expression in DLBCL was probably associated with the MYC‐oncogenic effect regardless of MYC rearrangements.^26^ Given the above discoveries, it is not surprising that MYC protein expression can still be displayed in the absence of detectable MYC rearrangement.

Many studies have demonstrated that MYC protein overexpression is relevant to inferior outcomes within the entire DLBCL cohort, in accordance with its central role in the regulation of thousands of genes.^6^, ^21^, ^27^, ^28^ Consistent with the above conclusions, this study also confirmed that MYC protein overexpression was associated with inferior OS and PFS and exhibited a worse response to treatment in patients with bulky mass DLBCL. Moreover, MYC protein overexpression retained its significant prognostic value after adjusting for the IPI score. We further investigated the survival outcomes of MYC protein expression status in different subgroups. Our study found that MYC overexpression was a factor for poor prognosis only in the subgroups with IPI score of 3–5 and Ann Arbor stage 3–4 but not in the subgroups with IPI score of 0–2 and Ann Arbor stage 1–2. This suggests that MYC protein expression is an excellent prognostic indicator for bulky mass DLBCL with high‐risk factors. Another interesting finding was that MYC overexpression was related to poor OS and PFS in the GCB subtype but not in the non‐GCB subtype, which was in contrast to some studies of DLBCL cohorts.^6^ Burkitt's lymphoma (BL) harbors a dysregulation of MYC, and the majority of its COO subtype is the GCB subtype.^2^, ^3^ Dave et al. showed that one DLBCL case had a probability of 66% for the diagnosis of BL, which might represent a rare genetic overlap between DLBCL and BL.^2^ Therefore, we speculate that bulky mass DLBCL with both MYC overexpression and the GCB subtype might have similar biological specificity to BL. In conclusion, MYC overexpression is a poor predictor for bulky mass DLBCL patients, especially those with high‐risk factors and the GCB subtype.

Regarding the effect of other biological parameters in patients with bulky mass DLBCL, this study found that the MYC/BCL2 DE status failed to confer a significant prognostic difference in OS and PFS, contrary to several studies of an entire DLBCL cohort.^5^, ^6^, ^7^, ^8^, ^9^ The most likely reasons for this discrepancy were that this study targeted the bulky mass cohort, with heterogeneity that differed from the entire DLBCL cohort. In addition, we found that DH also had no impact on survival in bulky mass DLBCL patients, but this might not be a statistically significant conclusion due to the insufficient amount of data and therefore should be treated with caution.

The inferior response to traditional therapy and poor survival outcomes highlight that alternative therapeutic strategies for bulky mass DLBCL patients are warranted. Therefore, we further explored the impact of different treatment strategies on the prognosis of bulky mass DLBCL. Several studies have confirmed the prognostic effect of dose‐intensified immunochemotherapy regimens on different subgroups of DLBCL, and the conclusions were mixed. Some studies have shown that R‐EPOCH or other dose‐intensified regimens improved the prognosis of certain DLBCL subgroups, such as DE, DT/TH, high‐risk young, or high Ki‐67 expression subgroups.^29^, ^30^, ^31^, ^32^, ^33^ For instance, Dunleavy et al. showed that DA‐EPOCH‐R produced durable remission in patients with MYC‐rearranged aggressive B‐cell lymphomas.^34^ However, the Alliance/CALGB 50303 study identified that DA‐EPOCH‐R failed to show its advantages over R‐CHOP in OS and PFS, including the high‐risk IPI subgroup and other subgroups, and grade 3/4 treatment‐related toxicity was more common in the DA‐EPOCH‐R group.^35^ Our study found that a dose‐intensive regimen such as R‐EPOCH did not improve the prognosis of patients with bulky mass DLBCL or with MYC overexpression. Moreover, our study found that surgery to relieve tumor burden prior to first‐line induction therapy and autologous hematopoietic stem cell transplantation did not improve the outcomes of patients with bulky mass DLBCL. In recent years, an increasing number of new drugs have been explored for application in DLBCL. Grzegorz et al. showed that the addition of lenalidomide to R‐CHOP did not improve prognosis in the bulky mass subgroup compared to R‐CHOP.^36^ The POLARIX study also demonstrated that polatuzumab vedotin, an antibody‐drug conjugate targeting the B‐cell surface antigen receptor CD79b, in combination with R‐CHP did not present a clear benefit in patients who had bulky disease compared to R‐CHOP.^37^ By contrast, our study indicated significant improvements in OS and PFS among patients with bulky mass DLBCL who received consolidation RT to residual disease after induction therapy. Further subgroup analysis also showed that RT improved OS and PFS in the MYC overexpression subgroup. Tzankov et al. also showed that RT improved the survival of DLBCL, and this improvement was more profound in patients with MYC deregulation.^38^ This further illustrated that RT may overcome MYC‐related treatment resistance. Many studies have also confirmed the positive role of RT in DLBCL, particularly in bulky mass populations.^10^, ^16^, ^17^, ^18^

We acknowledge that this study had some limitations. Clinical information obtained by retrospective retrieval of medical records was not entirely complete, and there was selection bias. This study was conducted at a single center. Despite these limitations, the prognostic value of MYC protein expression proposed in this study was assessed in a relatively large cohort, and this study was the first to target the bulky mass population. FISH technology is laborious and fails to provide any information about deregulation at the gene transcriptional and translational levels. MYC protein expression determined by immunohistochemistry is readily performed in most laboratories and represents a promising tool for stratifying risk in daily practice. Therefore, its value as a predictive marker should not be underestimated.

In conclusion, this study has shown that MYC overexpression is a poor predictor for bulky mass DLBCL patients, and its adverse prognostic effect is more pronounced in the high‐risk population and those with the GCB subtype. Consolidation RT for residual disease after induction therapy improves outcomes for bulky mass DLBCL patients as well as those with MYC overexpression. Further investigation and prospective studies in patients with bulky mass DLBCL are warranted.

## AUTHOR CONTRIBUTIONS

**yanjie wang:** Conceptualization (equal); data curation (equal); formal analysis (equal); investigation (equal); methodology (equal); project administration (equal); resources (equal); software (equal); supervision (equal); validation (equal); visualization (equal); writing – original draft (equal); writing – review and editing (equal). **Donglin Liu:** Conceptualization (equal); data curation (equal); formal analysis (equal); investigation (equal); methodology (equal); project administration (equal); resources (equal); software (equal); supervision (equal); validation (equal); visualization (equal); writing – original draft (equal); writing – review and editing (equal). **Xudong Zhang:** Conceptualization (equal); funding acquisition (equal); supervision (equal). **Mingzhi Zhang:** Conceptualization (equal); funding acquisition (equal); supervision (equal). **Shenglei Li:** Data curation (equal); methodology (equal); validation (equal). **Xiaoyan Feng:** Data curation (equal); methodology (equal); validation (equal). **Meng Dong:** Data curation (equal); methodology (equal); validation (equal). **Shanshan Ma:** Data curation (equal). **Siyu Qian:** Data curation (equal). **Zeyuan Wang:** Data curation (equal). **Yue Zhang:** Data curation (equal). **Pengyuan Wang:** Data curation (equal). **Shuhao Mei:** Data curation (equal). **Qingjiang Chen:** Conceptualization (equal); funding acquisition (equal); resources (equal); supervision (equal).

## CONFLICT OF INTEREST STATEMENT

The authors declare no conflict of interest.

## ETHICS STATEMENT

Informed consent for the collection of medical information was obtained from all patients. The present study was conducted according to the Helsinki declaration and was approved by the ethics committee of the First Affiliated Hospital of Zhengzhou University (Approval number: 2022‐KY‐0869‐001).

## Data Availability

The data that support the findings of this study are available upon request from the corresponding author.
